# Comparison of medical outcomes and health care costs at the end of life between dialysis patients with and without cancer: a national population-based study

**DOI:** 10.1186/s12882-019-1440-9

**Published:** 2019-07-16

**Authors:** Jui-Kun Chiang, Jean-Shi Chen, Yee-Hsin Kao

**Affiliations:** 1 Department of Family Medicine, Dalin Tzu Chi Hospital, Buddhist Tzu Chi Medical Foundation, 2, Minsheng Road, Dalin, 622 Chiayi, Taiwan; 2 Department of Nephrology, Tainan Municipal Hospital (Managed by Show Chwan Medical Care Corporation), 670 Chung-Te Road, Tainan, 701 Taiwan; 3 Department of Family Medicine, Tainan Municipal Hospital (Managed by Show Chwan Medical Care Corporation), 670 Chung-Te Road, Tainan, 701 Taiwan

**Keywords:** End-of-life care, Dialysis, Cancer

## Abstract

**Background:**

Palliative care has improved the quality of end-of-life (EOL) care and lowered the health care cost of cancer, and these benefits should be extended to patients with other serious illnesses including end-stage kidney disease. We evaluated the quality of EOL care, survival probabilities, and health care costs for dialysis patients in their last month of life.

**Methods:**

We conducted a population-based study and analyzed data from Taiwan’s Longitudinal Health Insurance Database, which contains claims information of patient medical records, health care costs, and insurance system exit dates (our proxy for death between 2006 and 2011).

**Results:**

Data of 1177 adult patients who died of chronic hemodialysis or peritoneal dialysis were investigated. The mean age of these patients was 69.7 ± 11.9 years, and 585 (49.7%) were women. Some patients with dialysis received cardiopulmonary resuscitation (66.9%), died in a hospital (65.0%), or were admitted to an intensive care unit (51.0%) in the last month of life. We further classified these patients into two groups, namely dialysis with cancer (DC) (*n* = 149) and dialysis without cancer (D) (*n* = 1028). Only 19 dialysis patients received palliative care, and the proportion of patients receiving palliative care was higher in the DC group than in the D group (11.4% vs. 0.2%). The mean health care costs per person during the final month of life was similar between the DC and D groups (USD 2755 ± 259 vs. USD 2827 ± 88). Multivariate logistic regression showed that the DC group had lower odds of receiving cardiopulmonary resuscitation (CPR) (OR: 0.39, CI = 0.26–0.56, *p* < 0.001) procedures, higher odds of longer hospital stays than the third quartile (> 25 days) (OR: 1.52, CI = 1.01–2.29, *p* = 0.0046), and higher odds of being hospitalized more than once (OR: 2.26, CI = 1.42–3.59, *p* = 0.001) than the D group in the last month of life after adjustments.

**Conclusions:**

DC patients received hospice care more frequently, received CPR less frequently, and had similar health care costs. DC patients also had a higher risk of a hospital stay that lasted more than 25 days and more than one hospitalization compared with D patients in the final month of life.

**Electronic supplementary material:**

The online version of this article (10.1186/s12882-019-1440-9) contains supplementary material, which is available to authorized users.

## Background

Worldwide, the population of dialysis patients has been increasing continuously for the past three decades [1, 2]. Factors that might contribute to the increase are the rapidly aging global population and diseases, such as diabetes and hypertension, which increase the risk of chronic kidney diseases, resulting in an increase in the need for dialysis [1, 3]. Taiwan reported the highest number of treated end-stage renal disease (ESRD) patients at 3392 per million population (PMP) in 2016, followed by Japan at 2599 PMP and the United States 2196 PMP [2]. With advancements in dialysis and medical treatments, dialysis patient survival has increased in the past two decades [3]. The mortality of dialysis patients decreased by 26% from 2001 (187 per 1000 patient-years) to 2015 (138 per 1000 patient-years) in the United States [1]; however, the mortality did not significantly change between 2000 (113 per 1000 patient-years) and 2012 (120 per 1000 patient-years) in Taiwan [4]. Because Taiwan has the highest prevalence and incidence of dialysis worldwide, dialysis-related care has become a crucial public health and social issue.

Many dialysis patients have multiple comorbidities [5–7], and the patients reported feeling relatively dependent and less capable of participating in activities that they enjoyed. Therefore, these patients experienced an overall decline in functional status and quality of life [8]. A previous study reported that the prevalence of both physical and psychological symptoms was higher in patients with advanced chronic kidney disease than in those with advanced cancer in the last month of life [9]. The most common symptoms included fatigue, itchiness, drowsiness, dyspnea, poor concentration, pain, anorexia, edema, xerostomia, constipation, and nausea. The burden and severity of these symptoms increased in the last month of life [9].

End-of-life (EOL) care for dialysis patients is a crucial consideration for patients and their families, particularly when death is imminent. In the United States, the indicators of quality for patients with ESRD during the last 90 days of life are as follows: (1) number of hospital admissions, (2) days spent in the hospital, (3) intensive care unit (ICU) admissions, (4) intensive procedures received, such as cardiopulmonary resuscitation (CPR), (5) inpatient surgical procedures received, and (6) inpatient deaths [10]. In the current study, we modified the conditions and measures for dialysis patients in Taiwan. The selected period was the last month of life; the indicators of inpatient surgical procedures were omitted, while the other indicators of quality were retained. Because dialysis is a significant risk factor for mortality during surgery, including hip fracture and coronary artery bypass grafting, surgery was not considered appropriate for patients in the last month of life [11, 12]. Investigations of EOL care are usually accepted in the last month of life [13, 14]. Hence, in the current study, we explored the quality indicators of EOL care of dialysis patients in the last month of life.

Palliative care is an interdisciplinary, team-based approach to symptom management, provision of psychological support, and treatment decision-making for patients with serious illnesses and their families. Increasing evidence highlights that patients with cancer at the EOL receive numerous benefits from palliative care, including reduction in symptom burden [15], improvement in quality of life and mood [16, 17], better overall survival [17, 18], and improvement in caregiver outcomes [19]; furthermore, these benefits were also observed in patients receiving cancer treatments [20]. In Taiwan, the use of palliative care has gradually progressed since 1983, and the first palliative ward was established in 1990 [21]. In Taiwan, the palliative care system includes both inpatient palliative care, which is the predominant type, and home palliative care; both types of care are covered by Taiwan’s National Health Insurance (NHI) program. Since 2009, the scope of palliative care has been extended beyond cancer to eight serious illnesses, including ESRD. However, the majority of palliative care continues to focus on treating patients with advanced cancer. Dialysis patients have a higher risk of cancer than do the nondialysis patients [22]. The causes of the high risks of cancers in chronic dialysis patients have not been elucidated adequately. Plants containing aristolochic acid were believed to cause renal damage and urinary tract cancer and a possible reason for the increased incidence of urinary tract cancer. To qualify for palliative care, patients must discontinue life-extending treatments provided at their hospice diagnosis, which includes dialysis for those with a hospice diagnosis of ESRD. In Taiwan, most patients with ESRD choose dialysis until death because of the low financial barriers to health insurance access, convenient medical access, and improvements in dialysis care [23]. Thus, these patients are not eligible for palliative care unless they have another diagnosis such as cancer, which meets the criteria for palliative care. However, the use of palliative care significantly increased among patients with cancer in Taiwan since 2000 because of the NHI’s reimbursement of palliative services. Two national policies promoting palliative services for terminal cancer patients were implemented in 2011 [24].

A previous study showed that family-reported quality of EOL care was higher for cancer patients than for patients with ESRD. [25] Therefore, we compared the quality of EOL care between chronic dialysis patients with and without cancer. Furthermore, we explored health care costs in the last month of life for the two groups of dialysis patients. Taiwan has a unique full-coverage dialysis policy. In Taiwan, dialysis health care costs increased from USD 68.4 million in 2000 to USD 1.54 billion in 2011, which is an increase of 125.15% [4]. Medical expenses of dialysis were higher among outpatients than among inpatients. Accordingly, expenses of dialysis patients burden Taiwan’s NHI program tremendously.

The use of administrative databases to investigate patients with chronic kidney disease is becoming increasingly common [26–28]. The use of these databases has several advantages; they can be used for examining the outcomes of large, population-based patient samples, and researchers can use less time-consuming and less expensive research protocols that offer a “real world” picture to test the effectiveness of interventions and compare care and outcomes among health providers, thus facilitating quality improvement initiatives [29]. A previous study reported that more than 80% of the terminal cancer patients with renal failure received hemodialysis (HD), and almost 20% of terminally ill cancer patients in palliative care received HD. [30] In the current study, quality indicators of EOL care were compared between chronic dialysis patients with and without cancer and to examine survival and health care costs in the last month of life.

## Methods

### Data source

**Fig. 1:**
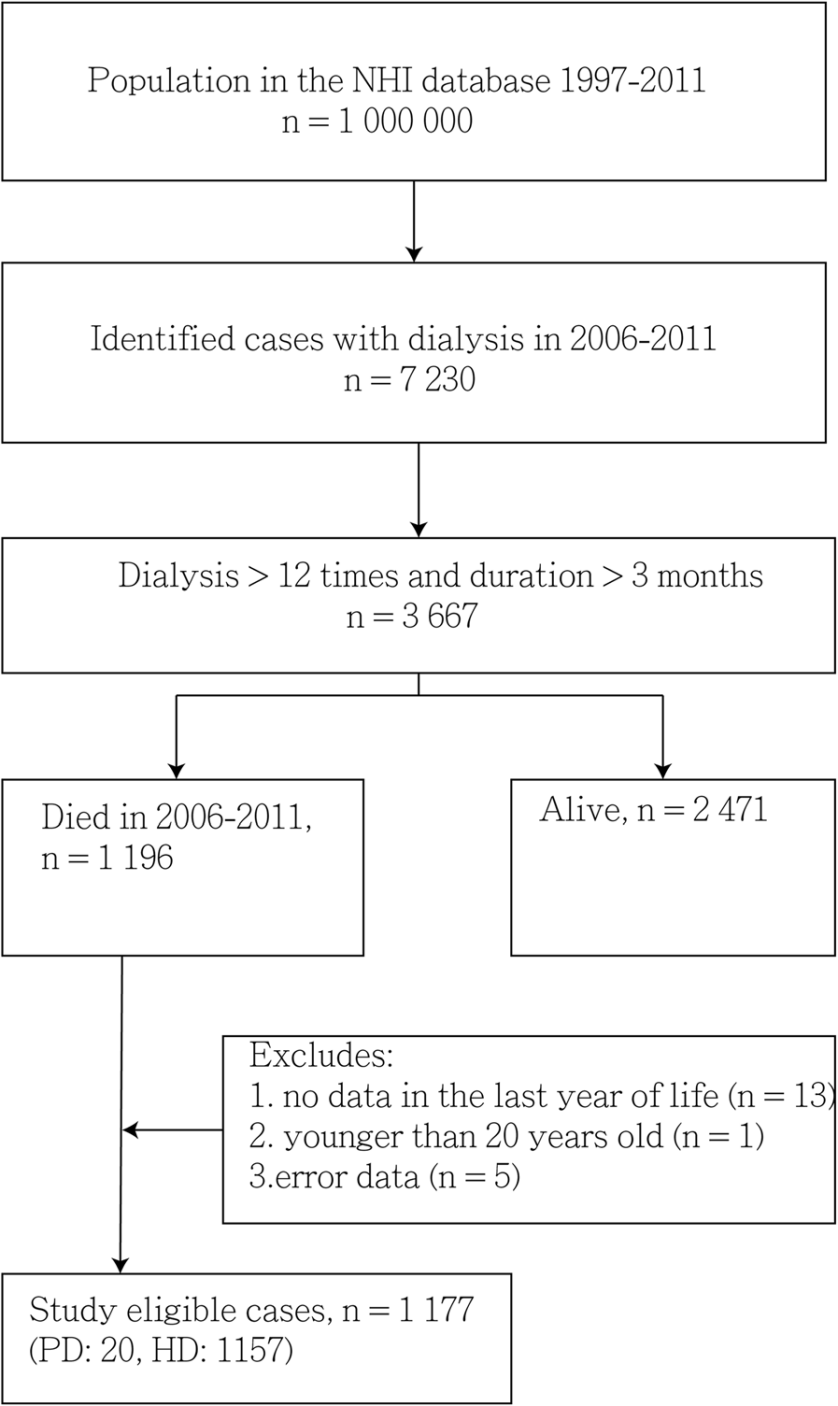
Flowchart of the study. Abbreviations: ICD-9-CM, International Classification of Diseases, Ninth Revision, Clinical Modification; CIC, catastrophic illness certificate; HD, hemodialysis; PD, peritoneal dialysis

In this nationwide population-based retrospective cohort study, we analyzed data obtained from the Taiwanese National Health Insurance Research Database (NHIRD). The NHI program, implemented in March 1995, is a single-payer health insurance system, which covered approximately 99.9% of the total population in 2012 [31]. The NHIRD, a nationwide representative database containing all original claims data of one million NHI beneficiaries from 1996 to 2012, is a randomized, systemic sample of the 23.32 million NHI enrollees. According to the NHIRD, patients with dialysis are designated as those having a catastrophic illness and are issued a catastrophic illness certificate. Patients with ESRD (ICD-9-CM code 585) who had received HD or peritoneal dialysis (PD) continuously for 3 months, with four dialysis procedures per month, were searched from the NHIRD. We used the ICD-9-CM code and charge master code to identify cases of HD and PD (Additional file 1). Our study cohort included patients who had received chronic dialysis from 2006 to 2011, with follow-up until December 2012 by using the Longitudinal Health Insurance Database 2000 (LHID2000), a subset of the NHIRD that contains all the original claims data of one million individuals randomly sampled from the NHIRD in 2000 (Fig. 1). We excluded the patients with a follow-up period of < 30 days and those who were younger than 20 years of age.

### Identification

#### Variables

Patient characteristics included age, sex, age at death, geographical location [32], urbanization level, and the final admission at a teaching hospital. Comorbid conditions listed in the Charlson comorbidity index (CCI) [33] and common comorbidities (e.g., cancer, diabetes, hypertension, stroke, myocardial infarction [MI], congestive heart failure [CHF], peripheral arterial occlusive disease [PAOD], chronic obstructive pulmonary disease [COPD], pneumonia, sepsis, and potassium imbalance) were identified using ICD-9-CM codes. A previous study reported that the most common cause of death in dialysis patients aged ≥75 years was withdrawal from dialysis, followed by cardiovascular diseases and infections [34]. Therefore, we added the comorbid conditions MI, CHF, PAOD, COPD, pneumonia, sepsis, and potassium imbalance (hyperkalemia or hypokalemia) to our analysis. To increase the validity of diagnosis of diabetes or hypertension, we only included the patients with three reported diagnoses of diabetes [35] or hypertension [36] in their medical claims data based on the ICD-9-CM codes for these disease entities.

#### Variable definitions

##### Chronic dialysis

Chronic dialysis was defined as receiving dialysis continuously for 3 months with more than four dialysis procedures per month from the claims data of the NHI [37].

##### Grouping of the dialysis patients

The patients whose medical records showed that they were on chronic dialysis and had cancer were categorized into the dialysis and cancer (DC) group, while the patients who were on chronic dialysis and did not have cancer were categorized into the dialysis (D) group. The NHIRD and catastrophic illness databases were used to identify patients with cancer between 2006 and 2011.

##### Billing encounter codes for palliative care

The codes for palliative inpatient care included P1101K, P1102A, P1103B, P1104K, P1105A, P1106B, 05601 K, 05602A, 05603B, 03001 KB, 03002AB, 03003BB, and 03004BB. The codes for palliative home care included 05312C, 05313C, 05314C, 05315C, 05323C, 05324C, 05325C, 05326C, 05327C, and 06314C. The codes for palliative outpatient care included 05311C, 05312C, 05313C, 05314C, 05315C, 05316C, 05326C, and 05327C.

##### CCI

We calculated the CCI scores by examining ICD-9-CM-based diagnoses and procedure codes recorded using the Deyo method. We subsequently applied the calculated indices to inpatient and outpatient claims reported by Klabundle et al. [38, 39].

##### Health care costs

We calculated each patient’s health care costs by adding the outpatient and inpatient service costs listed in his or her claims records. We converted these costs to USD based on the exchange rate between the US Dollar and the New Taiwan Dollar in 2006 (USD 1.00 = NTD 32.53).

##### Socioeconomic status of an individual

Socioeconomic status (SES) is a crucial factor for health care use [40, 41]. We classified the SES into three groups, namely low, moderate, and high in accordance with a previous study [42]. Those earning less than USD 922 per month, between USD 922 and USD 3074, and more than USD 3074 per month were categorized into the low, moderate, and high SES groups, respectively.

##### Aggressive EOL care in the last month of life

These five quality indicators of EOL dialysis care in the final month of life are as follows: more than one hospitalization, long hospital stays [longer than Q3 (25 days)], ICU admissions, CPR administration, and death in a hospital.

##### Hospital deaths

If the date of discharge for the last admission was the same as the date of death [43], the patient was considered to have died in the hospital.

### Statistical analysis

The distributional properties of continuous variables are expressed as the mean ± standard deviation or standard error and categorical variables as frequencies and percentages. The survival duration was defined as the duration from the date of diagnosis of dialysis to the date of death (in years). Survival probabilities were analyzed using the Kaplan–Meier method. Normality was examined using the Shapiro–Wilk test. In the univariate analysis, the two-sample *t* test, Wilcoxon rank-sum test, chi-squared test, and Fisher’s exact test were conducted to examine differences in the distributions of continuous variables and categorical variables between the DC and D groups.

We compared the patients’ demographic and clinical characteristics (Table 1), the primary causes of hospital admission in the last month of life (Table 2), and quality indicators in EOL dialysis care (Table 3) between the DC and D groups. All factors listed in Tables 1, 2, and 3 were included in the multivariate logistic regression models. A multivariate analysis was conducted using a stepwise variable selection procedure to determine the vital predictors of quality indicators during the last month of life. (Table 4) Collinearity between all the collected variables was checked.

**Table 1:** Comparison of demographic characteristics between dialysis patients with cancer (DC group) and without cancers (D group) during 2006–2011

Variables	Total	D group, n (%)	DC group, n (%)	*p* value
Total	1177	1028 (87.3%)	149 (12.7%)	
Gender				0.727
Female	585 (49.7%)	513 (49.9%)	72 (48.3%)	
Male	592 (50.3%)	515 (50.1%)	77 (51.7%)	
Age, years	69.7 ± 11.9	69.7 ± 12.1	69.9 ± 10.4	0.791
Survival (years, median)	2.63	2.63	2.52	0.300
CCI	4.7 ± 4.2	4.6 ± 4.0	5.4 ± 5.3	0.270
Diabetes	696 (59.1%)	636 (61.9%)	60 (40.3%)	< 0.001
Hypertension	908 (77.1%)	803 (78.1%)	105 (70.5%)	0.047
Stroke	339 (28.8%)	313 (30.4%)	26 (17.4%)	0.001
Socioeconomic status				
HSS	45 (3.8%)	36 (3.5%)	9 (6.0%)	0.165
MSS	378 (32.1%)	323 (31.4%)	55 (36.9%)	0.189
LSS	754 (64.1%)	669 (65.1%)	85 (57.0%)	0.067
Urbanization				
Urban	641 (54.5%)	562 (54.7%)	79 (53.0%)	0.725
Suburban	380 (32.3%)	329 (32.0%)	51 (34.2%)	0.575
Rural	156 (13.3%)	137 (13.3%)	19 (12.8%)	1
Teaching hospital in the last month of life	595 (54.2%)	521 (54.4%)	74 (52.5%)	0.717

*Abbreviations*: *CCI* Charlson co-morbidity index, *HSS* high socioeconomic status, *MSS* moderate socioeconomic status, *LSS* low socioeconomic status

**Table 2:** The primary causes of hospital admission in the last month for dialysis patients with cancer (DC group) and without cancer (D group) during 2006–2011

Variables	Total	D group n (%)	DC group n (%)	*p* value
Total	1177	1028 (87.3%)	149 (12.7%)	
Myocardial infarction	82 (7.0%)	77 (7.5%)	5 (3.4%)	0.083
Congestive heart failure	110 (9.3%)	103 (10.0%)	7 (4.7%)	0.035
PAOD	50 (4.2%)	48 (4.7%)	2 (1.3%)	0.078
COPD	52 (4.4%)	39 (3.8%)	13 (8.7%)	0.016
Pneumonia	229 (19.5%)	202 (19.6%)	27 (18.1%)	0.740
Sepsis	357 (30.3%)	306 (29.8%)	51 (34.2%)	0.294
Potassium imbalance^a^	46 (3.9%)	42 (4.1%)	4 (2.7%)	0.504

*Abbreviations*: *COPD* chronic obstructive pulmonary disease, *PAOD* peripheral arterial occlusive disease

^a^Potassium imbalance includes hyperkalemia or hypokalemia

**Table 3:** The comparison of the aggressiveness of care in the last month of life between dialysis patients with cancer (DC group) and without cancer (D group) during 2006–2011

Variables	Total	D group n (%)	DC group n (%)	*p* value
Total	1177	1028 (87.3%)	149 (12.7%)	
≥2 Hospitalizations	178 (15.1%)	141 (13.7%)	37 (24.8%)	0.001
Hospital stays (days)	13.1 ± 11.4	12.7 ± 11.4	15.4 ± 11.3	0.005
Hospital stays more than Q3 (25 days)	281 (23.9%)	234 (22.8%)	47 (31.5%)	0.023
ICU admission	600 (51.0%)	537 (52.2%)	63 (42.3%)	0.028
CPR	788 (66.9%)	718 (69.8%)	70 (47.0%)	< 0.001
Palliative care	19 (1.6%)	2 (0.2%)	17 (11.4%)	< 0.001
2006–2009	8 (0.7%)	1(0.1%)	7 (4.7%)	1
2010–2011	11 (0.9%)	1 (0.1%)	10 (6.7%)	
Dying in a hospital	765 (65.0%)	656 (63.8%)	109 (73.2%)	0.027
Cost^a^ (US$)	2818 ± 107	2827 ± 88	2755 ± 259	0.917

*Abbreviation*s: *ICU* intensive care unit, *CPR* cardiopulmonary resuscitation

^a^mean ± standard error

**Table 4:** The significant factors for the quality indicators by multivariate logistic regression for dialysis patients in the last month of life during 2006–2011 after adjustments

Variable	hospital stays > Q3 (25 days)	≥ 2 Hospitalizations	ICU	CPR	Dying in Hospital
Male vs. female	0.70 (0.52–0.94) (0.018)	–	–	–	–
Age	–	–	0.98 (0.97–0.99) (0.002)	0.98 (0.96–0.99) (< 0.001)	0.98 (0.97–1.00) (0.014)
DC group vs. D group	1.52 (1.01–2.29) (0.046)	2.26 (1.42–3.59) (0.001)	–	0.39 (0.26–0.56) (< 0.001)	–
Palliative care,	–	0.99 (0.18–5.58)	(0.01–0.85)	0.10 (0.01–0.85)	3.29 (0.35–30.5)
2006–2009		(0.993)	(0.035)	(0.035)	(0.295)
2010–2011		0.94 (0.66–1.34) (0.728)	0.84 (0.65–1.08) (0.179)	0.74 (0.56–0.98) (0.034)	1.13 (0.84–1.53) (0.423)
Admission days	–	1.04 (1.03–1.06) (< 0.001)	1.05 (1.04–1.06) (< 0.001)	1.02 (1.01–1.03) (0.002)	1.10 (1.08–1.11) (< 0.001)
Potassium imbalance^a^	0.06 (0.01–0.44) (0.006)	2.61 (1.20–5.65) (0.015)	–	–	–
CCI	1.04 (1.00–1.07) (0.046)	–	–	–	–
Nagelkerke’s R squared	0.154	0.209	0.197	0.120	0.335
Hosme-Lemeshow test	0.068	0.085	< 0.001	0.851	< 0.001

The values indicated: estimate (*p* value) (95%CI)

*Abbreviations*: *CCI* Charlson co-morbidity index, *CPR* cardiopulmonary resuscitation, *ICU* intensive care unit

^a^Potassium imbalance includes hyperkalemia or hypokalemia

## Results

**Fig. 2:**
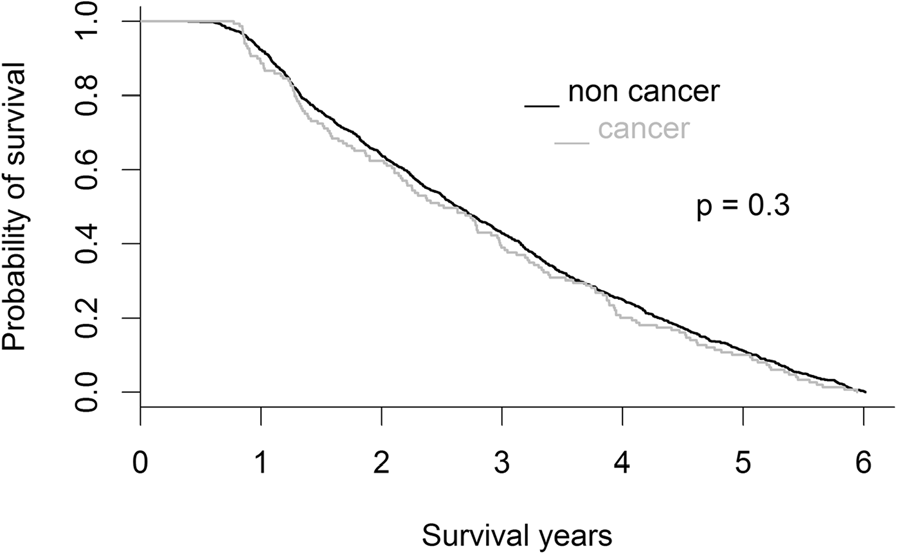
Survival probabilities of the dialysis patients with and without cancer

We enrolled 1177 adult patients on chronic dialysis (592 men and 585 women; ratio = 1.01:1) who died during 2006–2011. The patients usually exited from the insurance system after death, and the insurance system exit date was our proxy for death. The mean age of the patients was 69.7 ± 11.9 years. Among the chronic dialysis patients, 149 (12.7%) patients with cancer and 1028 (87.3%) were classified into the DC and D groups, respectively. Kidney and bladder (51, 34.2%), liver (35, 23.5%), and colon (24, 16.1%) cancer were the most common cancers in the DC group. Figure 1 presents the study design. Table 1 presents a comparison of the demographic characteristics of chronic dialysis patients between the DC and D groups. The percentage of diabetic patients in the DC group was lower than that in the D group (40.3% vs. 61.9%, *p* < 0.001). The percentage of patients with hypertension (70.5% vs. 78.1%, *p* = 0.047) and stroke (17.4% vs. 30.4%, *p* = 0.001) was also lower in the DC group than in the D group. The survival probabilities of dialysis patients between DC and D groups were not significantly different (*p* = 0.300) (Fig. 2). Table 2 presents a comparison of comorbid conditions in the last month of life between the DC and D groups. The DC group had a lower percentage of patients with congestive heart failure (4.7% vs. 10.0%, *p* = 0.034) and a higher percentage of patients with COPD (8.7% vs. 3.8%, *p* = 0.016) than did the D group.

Table 3 shows quality indicators of EOL dialysis care and health care cost comparisons between the DC and D groups during the last month of life. The DC group had significantly lower percentages of patients admitted to the ICU and patients who received CPR (42.3% vs. 52.2% [*p* = 0.028] and 47.0% vs. 69.8% [*p* < 0.001], respectively) than the D group. Furthermore, the DC group had a higher percentage of patients who were hospitalized more than once and had longer hospital stays (24.8% vs. 13.7% [*p* = 0.001] and 15.4 ± 11.3 vs. 12.7 ± 11.4 [*p* = 0.005], respectively) than the D group. The DC group had a higher percentage of hospital stays that were more than Q3 (> 25 days) (31.5% vs. 22.8%, *p* = 0.023) and deaths in hospital (73.2% vs. 63.8%, *p* = 0.027) compared with the D group. A higher percentage of cancer patients received palliative care than did those who did not have cancer (11.4% vs. 0.2%, *p* < 0.001). The mean days from patients receiving palliative care to death were 34.47 ± 60.36 days (median = 15 days). The mean health care costs per person during the final month of life of the patients of the DC group were approximately 2.5% less than those of the D group, but the difference was not statistically significant (USD 2755 ± 259 vs. USD 2827 ± 88, *p* = 0.917). We further calculated the health care costs between dialysis patients with and without palliative care in the last month of life. The mean health care costs per person during the final month of life for dialysis patients receiving palliative care was slightly less than those who did not receive palliative care, but it did not reach statistical significance (USD 1967 ± 55 vs. USD 2832 ± 108, *p* = 0.786).

Significant factors for the five quality indicators of EOL care were explored through multivariate logistic regression (Table 4). The independent factors listed in Tables 1, 2, and 3 were included in these procedures. The DC group had higher odds of hospital stays exceeding the third quartile (> 25 days in this study) (OR = 1.52, 95% CI = 1.02–2.29, *p* = 0.046), higher odds of more than one (≥2) hospitalization (OR = 2.26, 95% CI = 1.42–3.59, *p* = 0.001), and lower odds of receiving CPR (OR = 0.39, 95% CI = 0.26–0.56, *p* < 0.001) than the D group after adjustment for demographic variables (e.g., history of hypertension, high SES, living in northern Taiwan, living in a suburban area, and receiving service in the teaching hospital in the last month of life) and the primary causes of hospital admission in the last month of life (e.g., myocardial infarction, congestive heart failure, PAOD, pneumonia, and sepsis) (listed in Additional file 2) (Table 4). The DC and D groups did not differ significantly in the percentage of patients receiving ICU care and dying in the hospital. In the current study, we found 20 patients (1.7%) who had received PD, and only 1 case belonged to DC group. The subgroup analysis for patients with only HD was similar to above results.

## Discussion

In this study, a high percentage of dialysis patients received CPR (66.9%), died in a hospital (65.0%), and were admitted to an ICU (51.0%) in the last month of life, but a low percentage of patients received palliative care (1.6%). Other novel findings were that patients in the DC group received fewer intensive treatments, such as CPR, but had higher odds of long hospital stays (more than the third quartile or > 25 days in this study) and of more than one hospitalization in the last month of life after adjustments than did those in the group. Aggressive treatments, such as CPR and admission into the ICU in the last month of life, were critical issues concerning EOL care. In 2014, the percentage of elderly patients with ESRD in the United States who received CPR and were admitted into the ICU within the last 90 days of life was 34 and 62%, respectively [10]. We found that the DC patients received fewer CPR procedures than did the D patients. One explanation could be that national policies that promote palliative care results in a significant increase in palliative care use and decrease in CPR treatments in advanced cancer patients [24]. In the current study, we found that a higher percentage of patients in the DC group than in the D group received palliative care. We suggest that policymakers should actively promote palliative care programs for chronic dialysis patients to improve EOL dialysis care.

Till date, no study specifically investigated EOL care in dialysis patients in Taiwan. A previous study reported that incorporating palliative care into the dialysis units affects near-EOL patterns, and dialysis patients receiving palliative care had fewer ICU admissions and CPR sessions than did those without palliative care [44]. In this study, we found that 15.1% of dialysis patients had more than one hospitalization, and the mean hospital stay was 13.1 ± 11.4 days in the last month of life. In the United States, the overall percentage of hospital admissions among elderly patients with ESRD within the last 90 days of life was 83.4%, and the median number of times the patients were hospitalized was two, with a median hospital stay of 17 days between 2000 and 2014 [10]. In this study, we found that the DC patients were more likely to be hospitalized, have more than one hospital admission, and have hospital stays that were longer than 25 days in the last month of life than the D patients (24.8% vs. 13.7%, 15.4 ± 11.3 vs. 12.7 ± 11.4 days, 31.5% vs. 22.8%, respectively). One reason for these differences was that DC patients were frequently hospitalized for acute problems and symptom treatments. Timeliness in providing patients and their families with appropriate symptom assessments and treatments might be a solution that warrants further research.

Place of death has become a key indicator of EOL care; most patients prefer death at home [45]. In 2014, in the United States, the percentage of elderly patients with ESRD who died in the hospital (and not at home) was 40% [10]. In this study, we found that a higher percentage of chronic dialysis patients (65.0%) died in a hospital than at home, and the DC patients were more likely to die in a hospital than the D patients. A possible explanation for this finding might be a different definition of “dying in a hospital” in this study. If the discharge date for the last hospital admission was the same as the date of death, the patient was considered to have died in the hospital.

In Taiwan, patients with terminal illnesses requiring palliative service must be transferred to a palliative ward in a hospital for consultation and evaluation. During their palliative care course, frequent hospital admissions, long hospital stays, as well as admission to a hospital for relieving pain and other symptoms and subsequent death in the hospital in the last month of life for are expected. Culture might be a factor influencing the decision to die at home or in hospital among terminally ill patients. For example, dying patients were commonly and formally discharged from hospital “against medical advice” with artificial respiratory support to allow patients to die at home in Taiwan [46]. Another possible reason might be that in traditional Chinese culture, death is a sensitive topic; any mention of death is considered sacrilegious and is avoided [47]. Therefore, dying patients were often admitted, which might explain the increase in hospitalizations in the last month of life.

In this study, only 1.6% dialysis patients received palliative care in their last month of life, which is lower than that in the United States, 13.5 to 27% from 2002 to 2014 [10, 48]. The proportion of the patients who received palliative care was higher in the DC group (11.4%) than in the D group (0.2%). An explanation could be the national policies promoting palliative care for patients with cancer and the significant increase in palliative care use [24]. Promoting palliative care for dialysis patients and developing close cooperation and communication between palliative care and dialysis departments to improve patient care are the next steps for policy providers in Taiwan. Another resolution can be to promote the Renal Physician Association 2010 practice guidelines that were updated to affirm patients’ rights to refuse dialysis [49].

Due to the implementation of the NHI system in 1995, the number of patients receiving maintenance dialysis has increased rapidly. A previous study reported that the huge economic burden associated with dialysis is detrimental to the quality of dialysis treatment. Achieving a balance between the economics and quality of care requires multidisciplinary cooperation [50]. In this study, we found that the mean health care cost per person of DC patients during the final month of life was approximately 2.5% lower but not significantly different from that of D patients. A previous study recommended that the clinical practice of palliative care and dialysis withdrawal might be helpful for countries with NHI systems [51]. These recommendations also helped to improve EOL dialysis care and health care costs. Although palliative care is covered by the NHI program in Taiwan, the barriers to palliative care and withdrawing dialysis for patients with advanced renal failure in Taiwan include patient-related factors (e.g., differences in goals and values, lack of efficient communication with physicians, and lack of advanced care planning); physician-related factors (e.g., uncertainty regarding medical ethics, unfamiliarity with the law and regulation, and fear of legal issues), and system-related factors (e.g., not addressing preferences for dialysis, transition of care, lack of community-based palliative care systems, family-centered decision-making model, and special cultural considerations). [51]

## Limitations

The quality indicators of EOL dialysis care have been challenged; therefore, we adopted and modified measures from the United States Renal System Data [10]. A previous study reported that scores on Kidney Disease Quality of Life-Short Form, which includes the subscales of physical functioning, emotional health, and social functioning, are strongly associated with 2-year mortality, independent of age and is a crucial quality indicator for EOL care [52]. This study has some limitations. First, the risk factors related to each quality indicator (e.g., clinical symptoms and signs, laboratory data, stages of cancer, and “do not resuscitate” orders) were not available from the administrative database. Second, the preferences of the patients and their family members treatment at the end of life may have affected some outcomes. A previous study reported that patients wanted to discuss advance care planning with their family rather than physicians. [53] Another previous study showed that the current EOL care failed to meet the needs of patients with advanced CKD; [54] future research is warranted to investigate the effectiveness of advance care planning for dialysis patients in Taiwan. Third, patients who received dialysis for < 3 months and < 12 times were excluded from this study because we wanted to eliminate the possibility of including patients with acute renal failure or renal failure patients with terminal illnesses. Fourth, in September 2009, the bureau of the NHI amended the fee-charging standard from patients with cancer to patients without cancer but with terminal illness, including ESRD. Hence, dialysis patients who received palliative care were limited before September 2009. Fifth, the results were not extended to PD patients in the current study, and we were unable to measure whether a patient received a kidney transplant. Sixth, this is a study of decedents, which provides valuable insights into EOL care, but is limited due to the non-prospective identification of patients (i.e., health care providers do not know which patients is going to die). Seventh, the information of withdrawal from dialysis was not available in the claims data. Finally, the retrospective design of this study was also a potential limitation.

## Conclusions

This study indicated that a high proportion of dialysis patients received life-sustaining treatments (e.g., CPR treatments and ICU admissions) and died in a hospital, which might indicate that the patients and their families had unmet needs. We recommend that future studies investigate how to mitigate the suffering and distress of dialysis patients during EOL. Long hospital stays and frequent hospitalizations warrant further investigation. We suggest that policymakers improve accountability in EOL dialysis care and actively promote palliative care programs.

## Additional files

Additional file 1: ICD-9-CM code and charge master codes to identify HD and PD cases. (DOC 23 kb)

Additional file 2: **Table S1.** Significant factors for the quality indicators by using multivariate logistic regression for dialysis patients in the last month of life during 2006–2011. (DOC 52 kb)

## Data Availability

The datasets are not publicly available but are available from the first author on reasonable request.

## Abbreviations

AUC: Area under the receiver operating characteristic curve
CCI: Charlson comorbidity index
CIC: Catastrophic illness certificate
COPD: Chronic obstructive pulmonary disease
CPR: Cardiopulmonary resuscitation
EOL: End-of-life
ESRD: End-stage renal disease
HSS: High socioeconomic status
ICU: Intensive care unit
LSS: Low socioeconomic status
MSS: Moderate socioeconomic status
NHI: National Health Insurance
NHIRD: National Health Insurance Research Database
PAOD: Peripheral arterial occlusive disease
