# Supplementary material for: Comparison of medical outcomes and health care costs at the end of life between dialysis patients with and without cancer: a national population-based study
Source: BMC Nephrol. 2019 Jul 16;20:265. doi: 10.1186/s12882-019-1440-9 (PMC6636130; doi:10.1186/s12882-019-1440-9)
Supplement: Supplementary file 1 — ICD-9-CM code and charge master codes to identify HD and PD cases. (DOC 23 kb) [file 12882_2019_1440_MOESM1_ESM.doc]

**Additional file 1.** ICD-9-CM code and charge master codes to identify HD and PD cases.

Patients with ERSD (ICD-9-CM code 585) that received hemodialysis (HD) or peritoneal dialysis (PD). Then we used charge master codes in NHIRD to identify HD and PD. The charge master code for HD included 58001C, 58002C, 58003C, 58007C, 58014C, 58018C, 58019C, 58020C, 58021C, 58022C, 58023C, 58024C, 58025C, 58027C, and 58029C. The charge master codes for PD included 58009B, 58010B, 58012B, 58011C, 58017C, and 58028C.
