# Supplementary material for: Comparison of medical outcomes and health care costs at the end of life between dialysis patients with and without cancer: a national population-based study
Source: BMC Nephrol. 2019 Jul 16;20:265. doi: 10.1186/s12882-019-1440-9 (PMC6636130; doi:10.1186/s12882-019-1440-9)
Supplement: Supplementary file 2 — Table S1. Significant factors for the quality indicators by using multivariate logistic regression for dialysis patients in the last month of life during 2006–2011. (DOC 52 kb) [file 12882_2019_1440_MOESM2_ESM.doc]

**Additional file 2: Table S1. The significant factors for the quality indicators by multivariate logistic regression for dialysis patients in the last month of life during 2006-2011**

| Variable | hospital stays > Q3 (25 days) | ≥ 2 Hospitalizations | ICU | CPR | Dying in Hospital |
| --- | --- | --- | --- | --- | --- |
| Intercept | -1.35 | -3.22 | 0.09 | 1.79 | -0.03 |
| Male vs. female | 0.70(0.52-0.94)  (0.018) | - | - | - | - |
| Age | - | - | 0.98(0.97-0.99)  (0.002) | 0.98(0.96-0.99)  (<0.001) | 0.98(0.97-1.00)  (0.013) |
| With cancer | 1.52(1.01-2.29)  (0.046) | 1.95(1.20-3.17)  (0.007) | - | 0.44(0.30-0.65)  (<0.001) | - |
| Palliative care | ~~-~~ | 2.99(1.01-8.87)  (0.048) | 0.04(0.01-0.30)  (0.001) | 0.10(0.02-0.44)  (0.002) | 9.44(1.21-73.94)  (0.033) |
| Admission days | - | 1.04(1.03-1.06)  (<0.001) | 1.05(1.04-1.06)  (<0.001) | 1.02(1.01-1.03)  (0.002) | 1.10(1.08-1.13)  (<0.001) |
| Myocardial Infarction | 0.34(0.15-0.76)  (0.010) | 2.13(1.14-3.97)  (0.018) | 5.81(3.24-10.40)  (<0.001) | 2.94(1.55-5.57)  (0.001) | 3.46(1.86-6.45)  (<0.001) |
| Sepsis | 0.35(0.25-0.51)  (<0.001) | 3.93(2.74-5.65)  (<0.001) | 2.63(1.98-3.50)  (<0.001) | - | 3.30(2.32-4.68)  (<0.001) |
| Congestive Heart Failure | 0.30(0.14-0.65)  (0.002) | 2.23(1.33-3.74)  (0.002) | - | - | 1.84(1.08-3.12)  (0.024) |
| PAOD | 0.26(0.08-0.87)  (0.029) | - | - | - | - |
| Pneumonia | - | 1.84(1.25-2.72)  (0.001) | - | - | 1.54(1.02-2.31)  (0.038) |
| Potassium imbalance* | 0.06(0.01-0.44)  (0.006) | 2.46(1.13-5.37)  (0.024) | - | - | - |
| Hypertension | 1.63(1.12-2.37)  (0.011) | - | - | 1.46(1.06-1.99)  (0.019) | - |
| CCI | 1.04(1.00-1.07)  (0.046) | - | - | - | - |
| HES | - | 0.11(0.01-0.86)  (0.035) | - | - | -- |
| Northern Taiwan area | - | 0.66(0.45-0.96)  (0.029) | - | - | - |
| Suburban | 0.71(0.51-0.99)  (0.041) | - | - | - | - |
| Teaching hospital, in the last month | 1.80(1.31-2.47)  (<0.001) | - | - | 1.62(1.24-2.12)  (<0.001) | 1.38(1.03-1.85)  (0.030) |
| Nagelkerke's R squared | 0.154 | 0.214 | 0.210 | 0.124 | 0.341 |
| Hosme-Lemeshow test | 0.068 | 0.015 | <0.001 | 0.756 | <0.001 |
| AUC | 0.725(0.691-0.759) | 0.775(0.740-0.810) | 0.731(0.703-0.760) | 0.677(0.643-0.710) | 0.808(0.779-0.837) |

The values indicated: estimate (*p* value) (95%CI).

Abbreviation: AUC, area under the receiver operating characteristic curve; CCI, Charlson co-morbidity index; CPR, cardiopulmonary resuscitation; HSS, high socioeconomic status; ICU, intensive care unit; PAOD, peripheral arterial occlusive disease.

* Potassium imbalance includes hyperkalemia or hypokalemia
